# How to Use Costs in Value-Based Healthcare: Learning from Real-life Examples

**DOI:** 10.1007/s11606-023-08423-w

**Published:** 2023-12-22

**Authors:** Esmée K. J. van der Poort, Martha Kidanemariam, Christopher Moriates, Margot M. Rakers, Joel Tsevat, Marielle Schroijen, Douwe E. Atsma, M. Elske van den Akker-van Marle, Willem Jan W. Bos, Wilbert B. van den Hout

**Affiliations:** 1 Department of Biomedical Data Sciences, Section of Medical Decision-Making, Leiden University Medical Center, Leiden, The Netherlands; 2 Department of Internal Medicine, Dell Medical School, University of Texas, Austin, TX USA; 3 Department of Medical Education, Dell Medical School, University of Texas, Austin, TX USA; 4 National eHealth Living Lab, Leiden University Medical Center, Leiden, The Netherlands; 5 Department of Public Health and Primary Care, Leiden University Medical Center, Leiden, The Netherlands; 6 Department of Medicine and ReACH Center, Joe R. & Teresa Lozano Long School of Medicine, University of Texas Health Science Center at San Antonio, San Antonio, TX USA; 7 Department of Internal Medicine, Section of Endocrinology, Leiden University Medical Center, Leiden, The Netherlands; 8 Department of Cardiology, Leiden University Medical Center, Leiden, The Netherlands; 9 Department of Internal Medicine, Section of Nephrology, Leiden University Medical Center, Leiden, The Netherlands; 10 Department of Internal Medicine, St. Antonius Hospital, Nieuwegein, The Netherlands

**Keywords:** value-based healthcare, costs, shared decision-making, continuous improvement, benchmarking

## Abstract

**Background:**

Healthcare organizations measure costs for business operations but do not routinely incorporate costs in decision-making on the value of care.

**Aim:**

Provide guidance on how to use costs in value-based healthcare (VBHC) delivery at different levels of the healthcare system.

**Setting and Participants:**

Integrated practice units (IPUs) for diabetes mellitus (DM) and for acute myocardial infarction (AMI) at the Leiden University Medical Center and a collaboration of seven breast cancer IPUs of the Santeon group, all in the Netherlands.

**Program Description and Evaluation:**

VBHC aims to optimize care delivery to the patient by understanding how costs relate to outcomes. At the level of shared decision-making between patient and clinician, yearly check-up consultations for DM type I were analyzed for patient-relevant costs. In benchmarking among providers, quantities of cost drivers for breast cancer care were assessed in scorecards. In continuous learning, cost-effectiveness analysis was compared with radar chart analysis to assess the value of telemonitoring in outpatient follow-up.

**Discussion:**

Costs vary among providers in healthcare, but also between provider and patient. The joint analysis of outcomes and costs using appropriate methods helps identify and optimize the aspects of care that drive desired outcomes and value.

## INTRODUCTION

Due to the continuing rise in healthcare expenditure,^1^ considering the costs of care is clearly relevant. Value-based healthcare (VBHC) is a management strategy that has been progressively implemented in healthcare to optimize value to the patient by considering both outcomes and costs. One of the fundamental ideas of VBHC is that healthcare organizations can redesign and incrementally improve care delivery by understanding how costs are related to outcomes.^2,3^ Yet, VBHC still lacks robust methods for the joint analysis of outcomes and costs required to understand what drives value.^4,5^ In practice, healthcare organizations do measure costs for business but do not routinely incorporate costs in decision-making on the value of care.^6–8^ This article provides guidance on how to use costs in VBHC delivery at different levels of the healthcare system by learning from real-life examples from the Netherlands.

The strength of VBHC is that it aims to optimize patient-relevant outcomes for every unit of currency spent.^9^ Attempting to optimize outcomes without other considerations could result in expending large amounts of scarce resources for a negligible improvement in outcomes, at the expense of more efficient uses. On the other hand, solely focusing on costs could mean skimping on healthcare services and cutting budgets at the expense of beneficial health outcomes for patients, resulting in lower quality of care. Here, costs should be distinguished from charges. Costs denote the resources required to deliver care whereas charges only reflect the (often arbitrary) price of healthcare services on medical bills.^8^ VBHC aims to include all costs surrounding the full cycle of care of the patient.^9^ Overall, value cannot be optimized when only outcomes or only costs are considered.

Since VBHC’s introduction in 2006,^10^ costs have not been included to the same extent as outcomes in VBHC implementation.^11^ In the Netherlands, the government has devised a 5-year national strategy for outcome-based healthcare to support transforming the Dutch health system towards VBHC.^12^ This strategy applies measured outcome data at different levels of the healthcare system: in shared decision-making (SDM) between patient and clinician,^11,13,14^ in continuous learning within an organization^15–17^, and in benchmarking among providers.^6,18,19^ Routine outcome measurement is also an important prerequisite for providers to obtain reimbursement for care provided under value- instead of volume-based payment methods.^20,21^ While the main focus of VBHC implementation has been the improvement of health outcomes, best practices for the use of costs at these different levels have emerged in the Netherlands.

Previously, Tsevat and Moriates have pointed out that VBHC should learn from cost-effectiveness analysis (CEA) when it comes to the joint analysis of outcomes and costs.^5^ CEA provides a formal assessment of the impact of medical interventions on costs and health effects to inform policy and clinical guidelines.^22,23^ In certain European contexts, CEA mostly uses quality-adjusted life years (QALYs) to aggregate outcomes in calculating an incremental cost-effectiveness ratio (ICER) to facilitate decision-making in the trade-off between outcomes and costs. There are apparent similarities between the value equation and the ICER used in CEA, but the value equation leaves many elements that are clearly outlined in CEA open to interpretation. These standardized components for CEA can also guide the joint analysis of outcomes and costs in VBHC delivery and provide a framework for analysis (Table 1). This highlights that costs can be included in VBHC delivery according to different perspectives. Below, we present three examples of specific applications in VBHC that have emerged in the Netherlands: SDM between patient and clinician, benchmarking among providers, and continuous learning within an organization.

**Table 1:** Standardized Components for the Joint Analysis of Outcomes and Costs in VBHC Delivery

Level	Patient	Provider	Among providers
Aim	Achieve high-value care based on what matters to patients	Incrementally improve the value of care	Learn from other healthcare providers
Application	Patient-clinician interaction, shared decision-making	Continuous learning and evaluation within an organization	Benchmarking within and among organizations
Perspective	Individual patient	Hospital, clinic, practice site, disease-specific	Hospital, disease-specific
Outcomes	Clinical outcomes, PROMs, PREMs	Clinical outcomes, PROMs, PREMs, quality, process, and performance indicators	Quality, process, and performance indicators
Costs	Premiums, out-of-pocket expenses, travel costs, patient time costs, lost earnings, and unemployment benefits	Resources used to deliver care, cost drivers	Resources used to deliver care, cost drivers
Valuation of costs	Patient financial toxicity surveys and other instruments	Costing methods such as TDABC	Costing methods such as TDABC
Comparator	Treatment options or “patients like me”	Own performance over time, between patient groups	Other providers
Time horizon	Short- and long-term	Cycles of 3–6 months	1 year
Standardization	None	Standardized outcome sets, e.g., by ICHOM ^24,25^	Scorecards or registries, e.g., Dutch Institute for Clinical Auditing ^19,26^
Analysis	Not aggregated	Both aggregated and not aggregated	Aggregated

*PROMs*, patient-reported outcome measures; *PREMs*, patient-reported experience measures; *TDABC*, time-driven activity-based costing; *ICHOM*, International Consortium for Health Outcomes Measurement

## SHARED DECISION-MAKING BETWEEN PATIENT AND CLINICIAN: DISCUSSING COSTS WITH PATIENTS WITH TYPE 1 DIABETES

There is growing evidence that costs matter to patients’ health choices and that patients benefit from discussing costs in SDM.^27^ To incorporate costs from the patient perspective, all costs that matter to the patient — not just the costs of care delivery — should be considered. According to CEA, costs from the patient perspective include premiums, out-of-pocket expenses, lost productivity costs (inability to work during or due to treatment or illness), and travel costs (to and from the care provider).^28^ As such, Leiden University Medical Center (LUMC), a university teaching hospital in the Netherlands, has adopted a structured consultation model in diabetes care.^29^ This model focuses on both patients’ life- and health-related factors, e.g., social context, which may involve financial hardship. It is standard practice to consider travel time and out-of-pocket costs in the patient-clinician interaction, especially since LUMC is a referral center for patients with rare types of diabetes, e.g., monogenic diabetes mellitus. Hence, patients can be referred from outside of the region, leading to long travel times and greater costs. An ongoing study at LUMC investigates the effects of a VBHC dashboard containing the consultation model.^30^ In that study, consultations between participating patients and their endocrinologist or diabetes nurse specialist were audio-taped and showed that cost-related issues have been regularly discussed even before implementation of the VBHC dashboard (see Box 1).

**Box 1** Quotes of types of patient costs discussed in yearly routine check-ups for patients with type 1 diabetes, recorded in an ongoing study (C = Clinician, P = Patient)

**Table:** 

***Coverage by health insurance and out-of-pocket costs*** C1: “Yes, because there are other statins out there.” P1: “Yes, that’s what the pharmacy said. Then I said: I am not a guinea pig. I have a medication that I can handle well and then I have to change. That there are other laws. I then spoke to the owner of the pharmacy, because I was quite angry. He said ‘just pay €162 per box.’” C2: “Yes, you have a BMI of 29.5, so you are not eligible for that reimbursement. […]Then you must have a BMI above 35.” […] P2: “But I could pay for it myself. If I avoid becoming blind, then I think it is worth having to pay €120 per month for it.” C3: “What we are talking about is called the Freestyle Libre [for HbA1c assessment, red.], that is only reimbursed with insulin [use]” P3: “That is quite expensive” C3: “That is quite expensive indeed” P3: “I thought, it costs approximately €60 per 2 weeks.” C3: “But if I think along with you, you said, ‛I would like to know my HbA1c more often.’” [….] C3: “What we could do is… A HbA1c assessment can also be done from home these days.”
***Productivity costs*** P4: “For me, the trigger remains work, stress. Everything unravels after that.” C4: “Stress is most important.” P4: “Yes work pressure, so I am looking to sell my office and enter into employment somewhere … But it remains that work is actually the biggest negative factor. It makes me do all the other negative things too. If that becomes too much, I will sleep less, less sleep will make me hungry, hunger will make me eat wrong, wrong food will make me fatter. Then I become lethargic again, I feel less like moving. And it goes down and the sugar goes up.” C4: “Yes, you’ve got it all figured out, that’s how it works.” P5: “Yes, I had a heart attack [date] and that has meant that I am temporarily on sick leave, so I do not experience any work stress. So the sugars have become a lot better. But I now also take into account what I eat, also because one of the risk factors is overweight.” C5: “No, and you said in the beginning: you don’t have work stress now. Now you are rehabilitating, but I assume that at some point you will go back to work.” P5: “Yes.” C5: “Are you going to approach that differently?” P5: “Yes, that really gave me a wake-up call. And stress is also one of the risk factors that I have to bring down […].”
***Travel costs*** P6: “I gave the lab results [from another lab] to the receptionist.” C6: “That seems okay, then we don’t have to repeat that.” P6: “Yes, because getting blood drawn here takes me 2½ hours; coming here from home, that seems like a waste of time.”

To optimize value at the individual level, both outcomes and costs from the patient perspective need to be considered in consultations, allowing patients and clinicians to explore and agree upon a fitting care plan. A systematic review by Witte and colleagues on financial toxicity after cancer diagnosis and treatment showed widespread recognition of financial burden, but measures that can assess patients’ financial burden are lacking.^31^ Recently, the Comprehensive Score for Financial Toxicity-Functional Assessment of Chronic Illness Therapy (COST-FACIT) was validated in a population of adults with diabetes and elevated HbA1c levels.^32^ More research on patient-centered tools that support cost considerations in the patient-clinician interaction is needed.

## BENCHMARKING AMONG PROVIDERS: OUTCOME AND COST INDICATORS IN BREAST CANCER CARE

Accurate costing in VBHC can be used to identify cost drivers, improve care pathways, evaluate those improvements, and facilitate benchmarking^33^ and, therefore, is essential in VBHC. Cost measurement always consists of two parts: measuring quantities of resources and determining the value of these resources in unit costs or prices.^34^ Benchmarking on costs can be challenging because it requires some form of uniform costing. One proposed approach for costing in VBHC is time-driven activity-based costing (TDABC),^35^ but TDABC is resource-intensive to implement.^36^ The Dutch Santeon group, a collaboration of seven independent large teaching hospitals, addresses this by using cost indicators — quantities of care activities that strongly impact the total costs of care — thus avoiding the need for uniform costing among hospitals.

Santeon benchmarks and learns from outcome, process, and cost indicators for specific medical conditions by incorporating semi-annual scorecards.^37^ In a consensus process with different groups of care professionals, the scorecard outcome and process indicators are selected based on existing outcome sets.^38^ Cost indicators are selected based on significant cost drivers in the integrated practice units.^6^ In the case of breast cancer care, the set of cost indicators includes length of stay in number of days, the proportion of primary breast-conserving operations performed as same-day surgeries (without overnight hospitalization), operating room time per patient in minutes, number of consultations per patient, diagnostic tests per patient, and number of expensive drug prescriptions.

In advance, all Santeon hospitals had expected that 85% of patients would undergo same-day surgery, whereas the percentage was in fact only 56%.^39^ Additional analyses showed that patients did not always know that same-day surgery was an option even though they might have preferred it. In addition, the high rate of morphine use in some hospitals resulted in nausea during hospitalizations. Since analysis of the scorecards, patient-clinician communication about outpatient treatment has improved and patients now receive a nerve block during surgery, rendering the use of morphine unnecessary. This resulted in 66% of patients undergoing same-day surgery. Here, fewer hospitalizations result in lower costs of care, which also drives better clinical and patient-relevant outcomes, together resulting in higher value.

## CONTINUOUS LEARNING WITHIN AN ORGANIZATION: EVALUATION OF TELEMONITORING IN MYOCARDIAL INFARCTION CARE

Through VBHC implementation, healthcare organizations aim to incrementally improve care by a process of continuous learning based on collected outcome and cost data. Outpatient follow-up in the care pathway for acute myocardial infarction (AMI) at LUMC was redesigned by implementing telemonitoring for blood pressure (BP) regulation at home. Patients received smart technology devices, including a BP monitor, electrocardiogram device, scale, and activity tracker to measure clinical and patient-reported outcomes at home. Office follow-up visits at 1 and 6 months were replaced with electronic consultations.^40^ LUMC initially evaluated the performance of the redesigned care pathway using a trial-based CEA from a department perspective with a threshold *p*-value < 0.05 for statistical significance. One year after its adoption, telemonitoring resulted in non-significant savings of €616 per patient (95% CI €133 to €1365; *p* = 0.11) and a statistically significant moderate gain of 0.05 QALYs (95% CI 0.01 to 0.09; *p* = 0.03) compared with usual care.^41^ In the care pathway for AMI, telemonitoring is the dominant strategy because it is slightly more effective and less expensive than usual care, supporting the decision to adopt telemonitoring in the care pathway.

**Figure 1:**
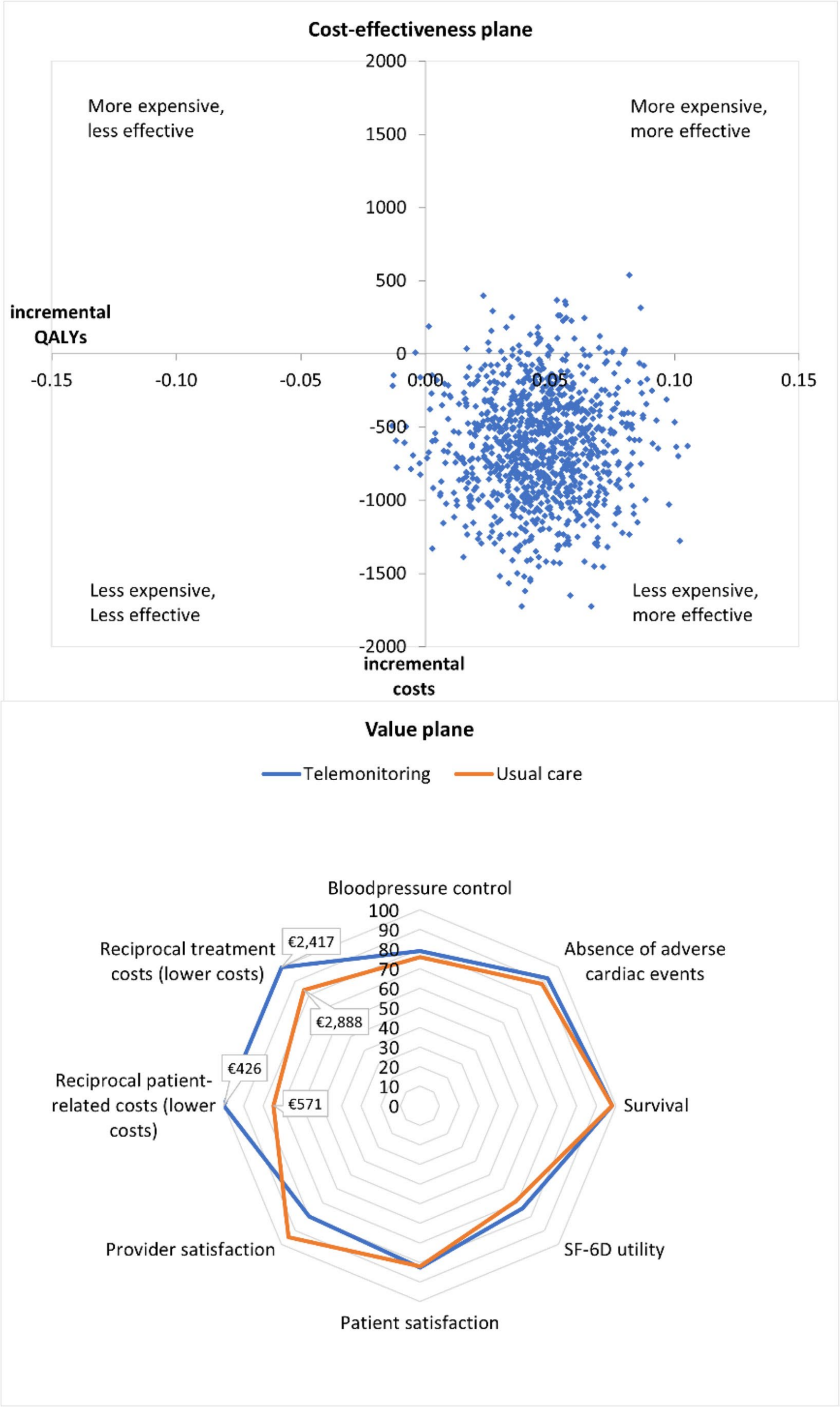
**A Cost-effectiveness plane from the trial-based CEA comparing telemonitoring with usual care. B Value plane presenting the assessed clinical and patient-relevant outcomes, experience measures, and costs. The costs are formatted as reciprocal costs so the outer value on the radar chart indicates favorable cost. Cost-effectiveness plane adapted from **^41^**. Copyright ©Roderick Willem Treskes, M Elske van den Akker-van Marle, Louise van Winden, Nicole van Keulen, Enno Tjeerd van der Velde, Saskia Beeres, Douwe Atsma, Martin Jan Schalij. Originally published in the Journal of Medical Internet Research (**https://www.jmir.org**), 25.04.2022.**

We used a radar chart analysis^42^ to compare the various outcomes and costs of telemonitoring and usual care according to VBHC principles and contrasted this to CEA (Fig. 1). First, the cost-effectiveness plane shows that BP telemonitoring is both less expensive and more effective than usual care, i.e., telemonitoring is dominant in CEA terminology. Alternatively, the radar chart shows costs from the organizational and patient perspectives, disaggregated outcomes, and experience measures assessed from the patient perspective (e.g., through patient-reported experience measures) and clinical perspective, including BP control, absence of adverse cardiac events, survival, health utility based on the SF-6D measure, and patient and provider satisfaction.^40^ For example, after 1 year, in the BP telemonitoring intervention group, 79% of patients had a systolic BP ≤ 139 mm Hg and a diastolic BP ≤ 89 mm Hg, as compared with 76% in the control group (*p* = 0.64). The radar chart shows lower costs and a slight increase in the SF-6D utility but worse provider satisfaction.

## DISCUSSION

In VBHC, there is no uniform criterion for the trade-off in these outcomes and costs. Presentation of separate outcome and cost domains allows viewers to form their own conclusions and helps clarify the outcome and cost domains that drive the value of changes in care delivery. The examples presented in this paper and the framework for analysis (Table 1) illustrate opportunities for standardizing the use of outcomes and costs in VBHC. First, the VBHC community should be aware of the study perspective when including costs. Costs vary among providers in healthcare, but also between provider and patient. The costs relevant to patients with type 1 DM as discussed in a SDM framework move beyond the scope of the resources required to deliver care that are used in benchmarking in the second example. The burden of costs to the patient can be captured through survey instruments, e.g., for financial toxicity, suitable for the patient-clinician interaction. Second, insight into which costs drive the value of care may help design alternative payment models. Last, a certain uniformity in cost measurement should be established among healthcare organizations to facilitate the use of costs in VBHC delivery. At present, the different costing methods used in healthcare organizations complicate comparing costs among providers.

However, the examples also show that considering outcomes and costs jointly in VBHC in a meaningful way is already possible. In the patient-clinician interaction, discussion of both outcomes that matter to the patient and patient-relevant costs will help improve the value of care tailored to the individual. Among providers, best practices can be shared and adopted through benchmarking on outcomes and cost drivers. Within healthcare organizations, providers can incrementally improve care delivery by assessing both outcomes and costs in continuous learning and evaluation. Moving forward, costs should be appreciated as a necessary and useful component of VBHC on all levels of the healthcare system.

## Data Availability

Data is available upon reasonable request if study participant consent was provided.

## References

[1] Organization WH. Global spending on health: rising to the pandemic’s challenges. Geneva; 2022. Licence: CC BY-NC-SA 3.0 IGO.

[2] **Porter ME, Lee TH.** The Strategy That Will Fix Health Care. Hardvard Business Review 91, 2013;(10):50-70.

[3] Porter ME (2008). Value-based health care delivery. Ann Surg..

[4] Walraven J, Jacobs MS, Uyl-de Groot CA (2021). Leveraging the Similarities Between Cost-Effectiveness Analysis and Value-Based Healthcare. Value in Health..

[5] Tsevat J, Moriates C (2018). Value-Based Health Care Meets Cost-Effectiveness Analysis. Ann Intern Med..

[6] **Mjåset C, Ikram U, Nagra NS, Feeley TW.** Value-based health care in four different health care systems. NEJM Catalyst. 2020.

[7] Cossio-Gil Y, Omara M, Watson C, Casey J, Chakhunashvili A, Gutierrez-San Miguel M (2022). The Roadmap for Implementing Value-Based Healthcare in European University Hospitals-Consensus Report and Recommendations. Value Health..

[8] Arora V, Moriates C, Shah N (2015). The Challenge of Understanding Health Care Costs and Charges. AMA J Ethics..

[9] Porter ME (2010). What Is Value in Health Care?. N Engl J Med.

[10] **Porter ME, Teisberg EO.** Redefining Health Care: Creating Value-Based Competition on Results. Boston: Harvard Business School Press; 2006.

[11] Vijverberg JRG, Daniels K, Steinmann G, Garvelink MM, Rouppe van der Voort MBV, Biesma D (2022). Mapping the extent, range and nature of research activity on value-based healthcare in the 15 years following its introduction (2006–2021): a scoping review. BMJ Open..

[12] **Larsson S, Clawson J, Howard R.** Value-Based Health Care at an Inflection Point: A Global Agenda for the Next Decade. NEJM Catalyst. 2023. https://catalyst.nejm.org/doi/full/10.1056/CAT.22.0332. Accessed 14 March 2023.

[13] Steinmann G, Delnoij D, van de Bovenkamp H, Groote R, Ahaus K (2021). Expert consensus on moving towards a value-based healthcare system in the Netherlands: a Delphi study. BMJ Open..

[14] Damman OC, Jani A, de Jong BA, Becker A, Metz MJ, de Bruijne MC (2020). The use of PROMs and shared decision-making in medical encounters with patients: An opportunity to deliver value-based health care to patients. J Eval Clin Pract..

[15] Greenhalgh J, Dalkin S, Gibbons E, Wright J, Valderas JM, Meads D (2018). How do aggregated patient-reported outcome measures data stimulate health care improvement? A realist synthesis. J Health Serv Res Policy..

[16] Prodinger B, Taylor P (2018). Improving quality of care through patient-reported outcome measures (PROMs): expert interviews using the NHS PROMs Programme and the Swedish quality registers for knee and hip arthroplasty as examples. BMC Health Serv Res..

[17] van Zijl F, Lohuis P, Datema FR (2022). The Rhinoplasty Health Care Monitor: Using Validated Questionnaires and a Web-Based Outcome Dashboard to Evaluate Personal Surgical Performance. Facial Plast Surg Aesthet Med..

[18] Katzan IL, Spertus J, Bettger JP, Bravata DM, Reeves MJ, Smith EE (2014). Risk adjustment of ischemic stroke outcomes for comparing hospital performance: a statement for healthcare professionals from the American Heart Association/American Stroke Association. Stroke..

[19] Kuhrij LS, Wouters MW, van den Berg-Vos RM, de Leeuw FE, Nederkoorn PJ (2018). The Dutch Acute Stroke Audit: Benchmarking acute stroke care in the Netherlands. Eur Stroke J..

[20] Hlavka JP, Lin PJ, Neumann PJ (2019). Outcome measures for oncology alternative payment models: practical considerations and recommendations. Am J Manag Care..

[21] Husaini M, Joynt Maddox KE (2020). Paying for Performance Improvement in Quality and Outcomes of Cardiovascular Care: Challenges and Prospects. Methodist Debakey Cardiovasc J..

[22] Sanders GD, Neumann PJ, Basu A, Brock DW, Feeny D, Krahn M (2016). Recommendations for Conduct, Methodological Practices, and Reporting of Cost-effectiveness Analyses: Second Panel on Cost-Effectiveness in Health and Medicine. JAMA..

[23] **Nederland Z.** Guideline for conducting economic evaluations in healthcare [in Dutch: Richtlijn voor het uitvoeren van economische evaluaties in de gezondheidszorg]. Diemen:Zorginstituut Nederland. 2015.

[24] **McNamara RL, Spatz ES, Kelley TA, Stowell CJ, Beltrame J, Heidenreich P, et al.** Standardized Outcome Measurement for Patients With Coronary Artery Disease: Consensus From the International Consortium for Health Outcomes Measurement (ICHOM). J Am Heart Assoc. 2015;4(5):e001767. 10.1161/JAHA.115.001767. 10.1161/JAHA.115.001767 PMC4599409 25991011

[25] Nano J, Carinci F, Okunade O, Whittaker S, Walbaum M, Barnard-Kelly K (2020). A standard set of person-centred outcomes for diabetes mellitus: results of an international and unified approach. Diabet Med..

[26] Busweiler LAD, Jeremiasen M, Wijnhoven BPL, Lindblad M, Lundell L, van de Velde CJH (2019). International benchmarking in oesophageal and gastric cancer surgery. BJS Open..

[27] Politi MC, Housten AJ, Forcino RC, Jansen J, Elwyn G (2023). Discussing Cost and Value in Patient Decision Aids and Shared Decision Making: A Call to Action. MDM Policy Pract..

[28] Garrison LP, Pauly MV, Willke RJ, Neumann PJ (2018). An Overview of Value, Perspective, and Decision Context-A Health Economics Approach: An ISPOR Special Task Force Report [2]. Value Health..

[29] Ruissen MM, Sont JK, van Vugt HA, Kunneman M, Rutten G, de Koning EJP (2022). Key Factors Relevant for Healthcare Decisions of Patients with Type 1 and Type 2 Diabetes in Secondary Care According to Healthcare Professionals. Patient Prefer Adherence..

[30] Rutten G, van Vugt HA, de Weerdt I, de Koning E (2018). Implementation of a Structured Diabetes Consultation Model to Facilitate a Person-Centered Approach: Results From a Nationwide Dutch Study. Diabetes Care..

[31] Witte J, Mehlis K, Surmann B, Lingnau R, Damm O, Greiner W (2019). Methods for measuring financial toxicity after cancer diagnosis and treatment: a systematic review and its implications. Ann Oncol..

[32] Patel MR, Zhang G, Heisler M, Song PXK, Piette JD, Shi X (2022). Measurement and Validation of the Comprehensive Score for Financial Toxicity (COST) in a Population With Diabetes. Diabetes Care..

[33] Leusder M, Porte P, Ahaus K, van Elten H (2022). Cost measurement in value-based healthcare: a systematic review. BMJ Open..

[34] **Drummond MF, Sculpher MJ, Stoddart GL, Torrance GW, Drummond M, Sculpher MJ, et al.** Methods for the economic evaluation of health care programmes. Fourth edition. ed. Oxford, United Kingdom: Oxford University Press; 2015.

[35] Kaplan RS, Porter ME (2011). How to solve the cost crisis in health care. Harv Bus Rev..

[36] Keel G, Savage C, Rafiq M, Mazzocato P (2017). Time-driven activity-based costing in health care: A systematic review of the literature. Health Policy..

[37] Santeon. ‘Better Together’ [in Dutch] https://santeon.nl/onze-aanpak/samen-beter/2022 [cited 2022 06–12]. Available from: https://santeon.nl/onze-aanpak/samen-beter/. Accessed 14-3-2023.

[38] Ong WL, Schouwenburg MG, van Bommel ACM, Stowell C, Allison KH, Benn KE (2017). A Standard Set of Value-Based Patient-Centered Outcomes for Breast Cancer: The International Consortium for Health Outcomes Measurement (ICHOM) Initiative. JAMA Oncol..

[39] **Lea Dijksman, Annermarie Haverhals, Maartje Wielders.** Beter Breast Cancer Care Through Collaboration. Utrecht: Santeon. 2017. https://santeon.nl/app/uploads/2022/08/Breast-cancer-2017-care-for-improvement.pdf. Accessed 14 March 2023.

[40] Treskes RW, van Winden LAM, van Keulen N, van der Velde ET, Beeres SLMA, Atsma DE (2020). Effect of Smartphone-Enabled Health Monitoring Devices vs Regular Follow-up on Blood Pressure Control Among Patients After Myocardial Infarction: A Randomized Clinical Trial. JAMA Netw Open..

[41] Treskes RW, van den Akker-van Marle ME, van Winden L, van Keulen N, van der Velde ET, Beeres S (2022). The Box-eHealth in the Outpatient Clinic Follow-up of Patients With Acute Myocardial Infarction: Cost-Utility Analysis. J Med Internet Res..

[42] Thaker NG, Ali TN, Porter ME, Feeley TW, Kaplan RS, Frank SJ (2016). Communicating Value in Health Care Using Radar Charts: A Case Study of Prostate Cancer. J Oncol Pract..

